# Molecular requirements for mammalian crinophagy highlight a key role for Ca^2+^-dependent Munc13-4

**DOI:** 10.21203/rs.3.rs-7349548/v1

**Published:** 2025-09-04

**Authors:** Muralidharan Mani, Declan J. James, Thomas F.J. Martin

**Affiliations:** University of Wisconsin-Madison; University of Wisconsin-Madison; University of Wisconsin-Madison

## Abstract

Secretory granules (SGs) in endocrine cells store and release peptide hormones with their turnover tightly controlled to maintain cellular hormone levels. We found that crinophagy, a specialized autophagy process, is the prevalent pathway that degrades older unused SGs in resting cells by SG-lysosome fusion. siRNA screening with a live cell assay for SG-lysosome merge identified SG components Rab27A, Munc13-4 and VAMP2 and lysosomal components PLEKHM1, HOPS subunits, and SNAREs STX7, STX8, and VTI1B required for docking SGs to and fusion with lysosomes. Munc13-4 is a central regulator of crinophagy that associates with many proteins that are functionally essential for the merge of SGs with lysosomes, and regulates the docking and fusion of SGs with lysosomes. SG-lysosome fusion was regulated by local or global calcium through binding and activation of Munc13-4. The findings reveal the critical docking/fusion machinery for mediating SG turnover in mammalian endocrine cells, and indicate how its dysregulation could impair hormonal and metabolic regulation.

## Introduction

Peptide prohormones are packaged into immature secretory granules (iSGs) in the Golgi of endocrine secretory cells, and mature hormones are stored in cytoplasmic secretory granules (SGs) for potential release via Ca^2+^-triggered exocytosis at the plasma membrane. However, the exocytic release of peptide hormones by stimulation is limited to a small fraction (1–10%) of SGs and the unused majority of SGs are degraded^1,2^. A balance between biogenesis and degradation must be achieved to maintain optimal hormone stores for their episodic regulated exocytic release. Disruptions in this balance contribute to endocrine, metabolic, and neurological disorders^3–5^.

Newly formed SGs undergo preferential regulated exocytosis whereas unused SGs are subject to age-dependent lysosomal degradation^6,7^. Several pathways for lysosomal SG degradation include macroautophagy, microautophagy, and crinophagy^8–12^. These have mainly been studied under stress conditions, and it is unclear which pathway mediates the basal homeostatic turnover of SGs. Macroautophagy and microautophagy involve the engulfment of SGs by phagophores and lysosomes, respectively^13,14^. In contrast, crinophagy was inferred from EM studies to involve the fusion of SGs with lysosomes^15,16^. Direct fusion with lysosomes would allow the degradation of unreleased SG contents such as aged peptide hormones but allow the recycling of SG membrane components for reuse. Increased crinophagy is associated with excessive peptide hormone stores, impaired SG exocytosis, or metabolic stress although the basis for this regulation is unknown^8,10,16,17^.

The molecular mechanisms that govern the heterotypic fusion of SGs with lysosomes in mammalian endocrine cells are not known. Studies identified mechanisms underlying glue granule degradation in *Drosophila* exocrine cells but whether these are conserved across cell types or species is unknown^18,19^. Here, we characterize SG-lysosome interactions in cultured endocrine cells and show that crinophagy is a membrane fusion process that preferentially degrades older SGs. A live-cell assay for SG-lysosome merge was used for siRNA screening to identify SG and lysosomal proteins essential for crinophagy and its regulation. Specifically, we identified essential membrane components on lysosomes as PLEKHM1, the HOPS complex, and SNAREs syntaxin7 (STX7), syntaxin8 (STX8), and vesicle transport through interaction with t-SNAREs homolog (VTI1B). Critical membrane components on SGs were the SNARE vesicle-associated membrane protein 2 (VAMP2), RAB27A, and its effector Munc13-4. We show that Munc13-4 localizes to SGs and functions as a critical tethering/priming factor that promotes SG docking onto lysosomes and regulates fusion. *In vitro* studies showed that Munc13-4 promotes the docking/fusion of SGs with lysosomes in the presence of Ca^2+^, and that crinophagy in cells is regulated by Ca^2+^. In summary, crinophagy in mammalian endocrine cells is a unique autophagic process, and Ca^2+^-dependent Munc13-4 is a critical driver of SG-lysosome fusion.

## Results

### Crinophagy mediates SG degradation in unstressed endocrine cells

**Figure 1:**
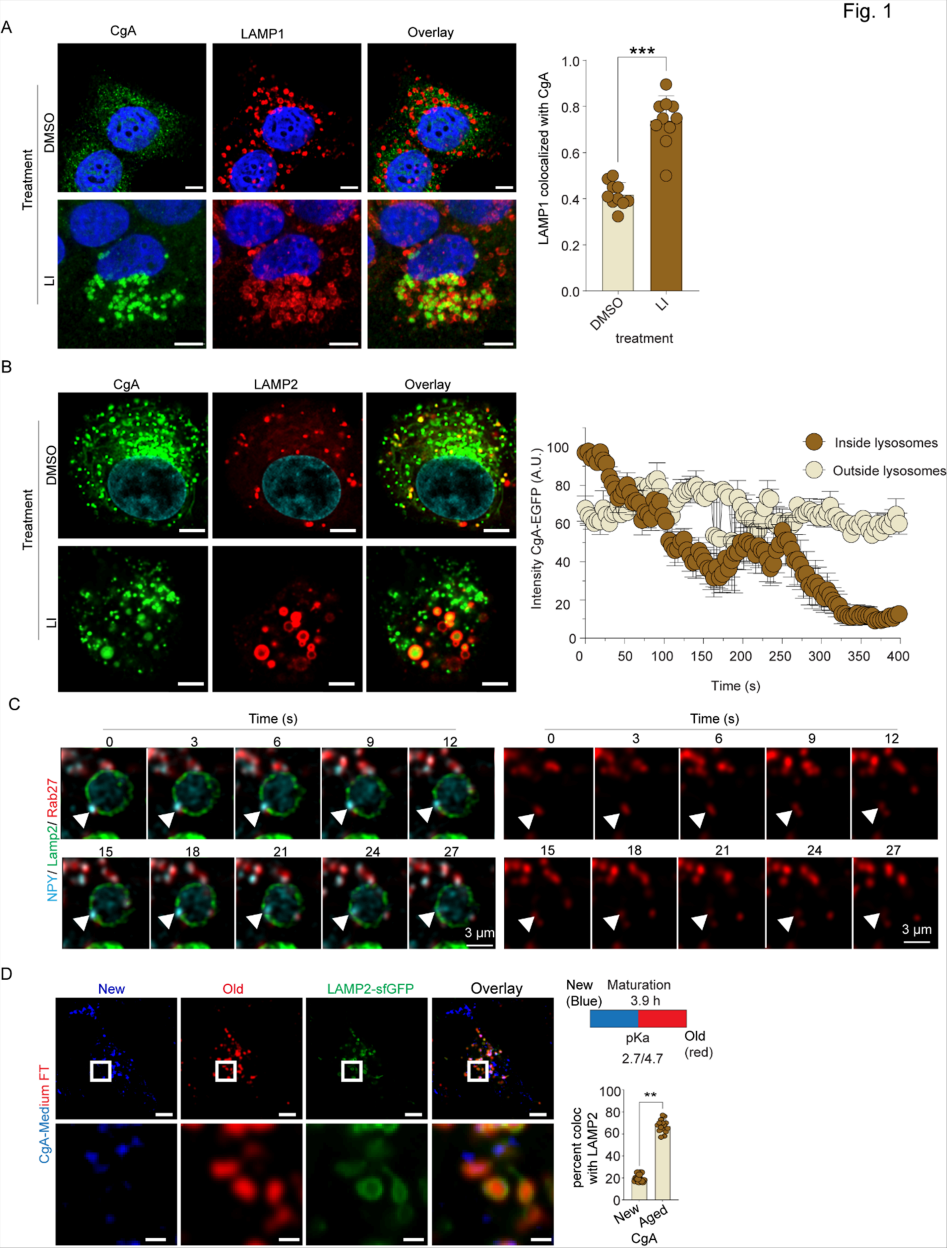
SG turnover in neuroendocrine cells. A. BON cells immunostained for CgA and LAMP1 in the presence of DMSO or Lysosomal inhibitor cocktail (LI) (pepstatin 1 μM and 1 μM E64D) for 4h. Colocalization was determined by calculating object Pearson’s correlation coefficient (n= > 15, mean ± SD, ***p <0.001) (Scale Bar, 5 μm). B. BON cells were transiently transfected with CgA-EGFP & LAMP2-mCherry for 16 hours and treated with LI for 4h. The graph was plotted by tracking the intensity of CgA both outside lysosomes and within the lysosomal compartments over time. C. NPY-halo BON cells were transiently co-transfected with LAMP2-mNeonGreen and mCherry-Rab27A. Prior to imaging, cells were labeled with Janelia Fluor^®^ 646 (JF646) to visualize NPY. Live-cell imaging was performed after a 16-hour incubation period with frames captured every 3 seconds. An arrowhead indicates NPY entry into lysosomes. Scale Bar, 3 μm. D. BON cells were transiently transfected with CgA-medium FT and LAMP2-sfGFP, and cells were imaged live after 16 h following transfection. The blue and red forms of CgA-medium FT are shown. Colocalization was determined by calculating the percentage of overlapping objects. (n= > 15, mean ± SD, **p <0.01) (Scale Bar, 7 μm).

We investigated crinophagy in neuroendocrine BON cells, a human cell line that exhibits characteristics of immature pancreatic β-cell progenitors^20^. The cells contain numerous large secretory granules (SGs) that store secretory peptides (chromogranins A-C, neurotensin) and biogenic amines (5-HT)^21,22^. Chromogranins are core components of SGs in neural and endocrine cells where they are released via Ca^2+^-triggered exocytosis^23^. Lysosome-associated membrane proteins (LAMP1 and LAMP2) are markers for lysosomal membranes^24^. To detect SG associations with lysosomes, we immunostained BON cells with specific antibodies to chromogranin A (CgA) and LAMP1 to examine colocalization by confocal microscopy. In untreated cells, colocalization of CgA-positive SGs with LAMP1 was limited (Fig. 1A, upper row). However, treatment with lysosomal inhibitors (LIs, 1 μM pepstatin A and E64D) significantly increased CgA detection within lysosomes and enhanced colocalization with LAMP1 (Fig. 1A, lower) by inhibiting CgA degradation. Consistently, Western blot analysis showed increased CgB levels in cell lysates following LI treatment without affecting CgB secretion (Figs S1A, B). These findings suggested there is a continuous flow of CgA from SGs to LAMP1-positive organelles at steady-state.

To observe SG content entry into lysosomes in real time, we generated BON cell lines expressing GFP-tagged CgA. Imaging CgA-EGFP with LAMP2-mCherry revealed a marked flux of SG content into lysosomes during LI treatment (Fig. 1B, lower). In SGs where content entered lysosomes (Fig. 1B inset, closed circles), the fluorescence of CgA-EGFP was quenched due to the low pH environment, while SGs remaining outside lysosomes were unquenched (Fig. 1B inset, open circles). Imaging individual SG-lysosome encounters showed that CgA-EGFP quenching occurred within ~ 15 seconds after SG contact with lysosomes indicating rapid entry of SG content into the lysosomal lumen (Fig. S1C). To determine the mode of entry of SG content into lysosomes, we expressed NPY-JF646 as SG content, Rab27A-mCherry as a SG membrane marker, and LAMP2-mNeonGreen as a lysosomal membrane marker for imaging SG-lysosome encounters (Fig. 1C). Rab27A and NPY co-localized on SGs including those that docked onto LAMP2-positive organelles (Fig. 1C, arrowhead). After docking, NPY in SGs entered the lysosomal lumen (Fig. 1C) whereas Rab27A remained at the lysosomal surface and later disappeared (Fig. 1C, right panels). The selective entry of NPY but not Rab27A into the lysosomal lumen indicates that SG NPY content entry was by fusion at sites of SG docking, which corresponds to a hallmark feature of crinophagy previously detected in EM studies^15,16^.

Only a subset of SGs delivered their contents into lysosomes. We hypothesized that these were older SGs as prior studies reported preferential degradation of aged SGs by lysosomes^7^. To investigate age-dependent degradation of SGs, we employed monomeric fluorescent timers (FTs) by fusing CgA to mCherry variants with distinct chromophore maturation rates without the need for chemical labeling or fixation^25^. After 18 hours, CgA-FT expression revealed two temporally distinct populations, blue (new) and red (aged) SGs (Fig. 1D). Aged SGs showed preferential association with LAMP2-positive lysosomes upon lysosomal inhibitor (LI) treatment, indicating the selective targeting of older SGs to lysosomes. Overall, these studies suggest that crinophagy is a major process for SG content turnover under resting conditions and provide additional evidence for SG-lysosome fusion.

### Macro- and microautophagy do not mediate SG degradation in unstressed endocrine cells

**Figure 2:**
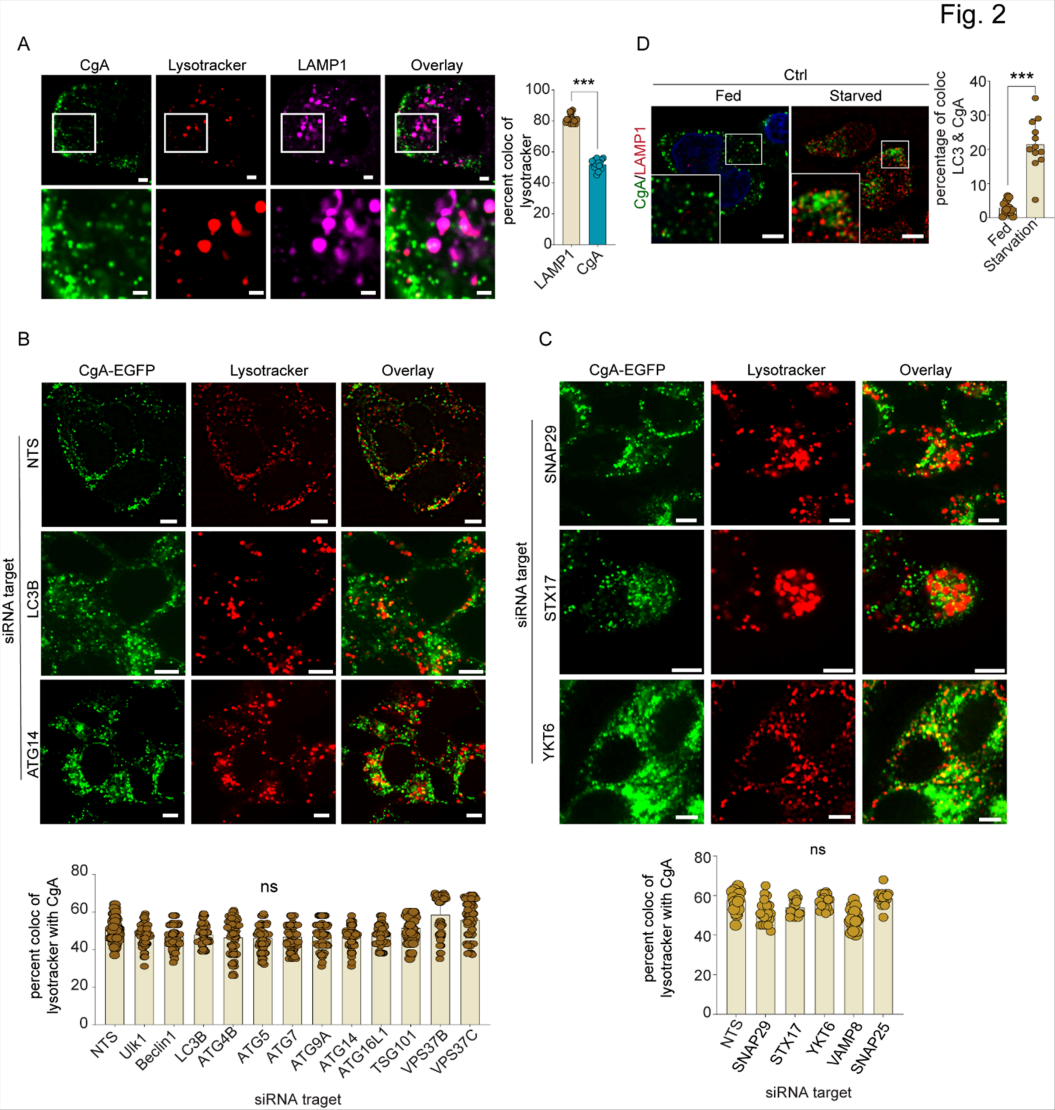
Macroautophagy is dispensable for SG degradation. A. BON cells expressing CgA-EGFP or LAMP2-sfGFP were treated with Lysotracker DND99. Colocalization analysis was calculated by determining the percentage of overlapping objects, based on the total number of Lysotracker objects that overlap with the total number of LAMP1 or CgA objects (n= > 15, mean ± SD, ***p <0.001) (Scale Bar, 5 μm, insert 1μm). B. CgA-GFP BON cells were treated with the indicated siRNAs for 72 hours. Colocalization of Lysotracker Red with CgA-GFP was quantitated following selective gene knockdown. C. Knockdown of autosomal and lysosomal SNAREs SNAP29, STX17 & YKT6 did not affect SG-lysosome merge. (B & C n = 35, N = 3, mean ± SD, ns). (Scale Bar, 5 μm). D. BON cells were cultured in either standard culture media (Fed) or media lacking glucose, amino acids, and FBS (Starved) for 4 hours, fixed, and stained with LC3 and CgA antibodies. The inserts depict magnified images from the same cells. Colocalization of endogenous LC3 with CgA (n= > 15, mean ± SD, ***p <0.001) (Scale Bar, 5 μm).

To investigate the proteins involved in SG turnover, we developed a live-cell assay for crinophagy using Lysotracker dyes in stable CgA-EGFP-expressing BON cells. Lysotracker dyes at low concentrations efficiently labelled acidic LAMP1-positive organelles^26^ but not the SGs in BON cells (Fig. 2A). The overlap between Lysotracker Red and CgA-EGFP was quantified to measure SG flux into lysosomes representing crinophagy activity.

Macroautophagy may mediate SG degradation under extreme conditions (e.g., starvation or stress)^9,11^, but its role in SG turnover in unstressed cells was unknown. We used siRNA screening to determine if SG-lysosome merge depends on macroautophagy-related proteins. Macroautophagy, a process involving autophagosome fusion with lysosomes to degrade cytoplasmic components, requires ATG proteins^27,28^. In CgA-EGFP-expressing BON cells, the knockdown of ATG proteins (Ulk1, Beclin-1, ATG4, ATG5, ATG7, ATG9, ATG14, and ATG16L1) showed no significant reduction in the overlap of CgA-EGFP with Lysotracker, indicating that SG-lysosome merge does not rely on these proteins in unstressed cells (Fig. 2B & S2A). Western blotting confirmed the efficient knockdown of the proteins (Fig. S2B). Similarly, the depletion of SNARE proteins (STX17, YKT6, SNAP29, and VAMP8) essential for autophagosome-lysosome fusion^29^, and tether proteins (EPG5, GRASP55, and BIRC6) required for macroautophagy^29^, did not affect SG-lysosome merge (Fig. 2C, S2D, S2E). In addition, colocalization studies of CgA with LC3B, an autophagy marker, in CgA-EGFP stable BON cells (Fig. 2D) and LC3-mApple-expressing cells (Fig. S2F), showed minimal overlap in unstressed conditions while starvation conditions moderately increased colocalization. The results suggest that macroautophagy does not play a major role in SG-lysosome merge under resting conditions.

Lysosomal engulfment by microautophagy was suggested as a possible basis for the degradation of Golgi-derived vesicles^30^ as well as SGs^8^. ATG proteins are also required for several forms of microautophagy^31^ as are lysosomal ESCRT proteins^30^. In addition to ATG proteins, we knocked down several ESCRT proteins without affecting SG-lysosome merge (Fig. 2B). This, in addition to the lack of observed lysosomal engulfment of dual-labeled SGs (Fig. 1C), suggests that microautophagy does not play a major role in the SG-lysosome merge in BON cells under resting conditions.

**Figure 3:**
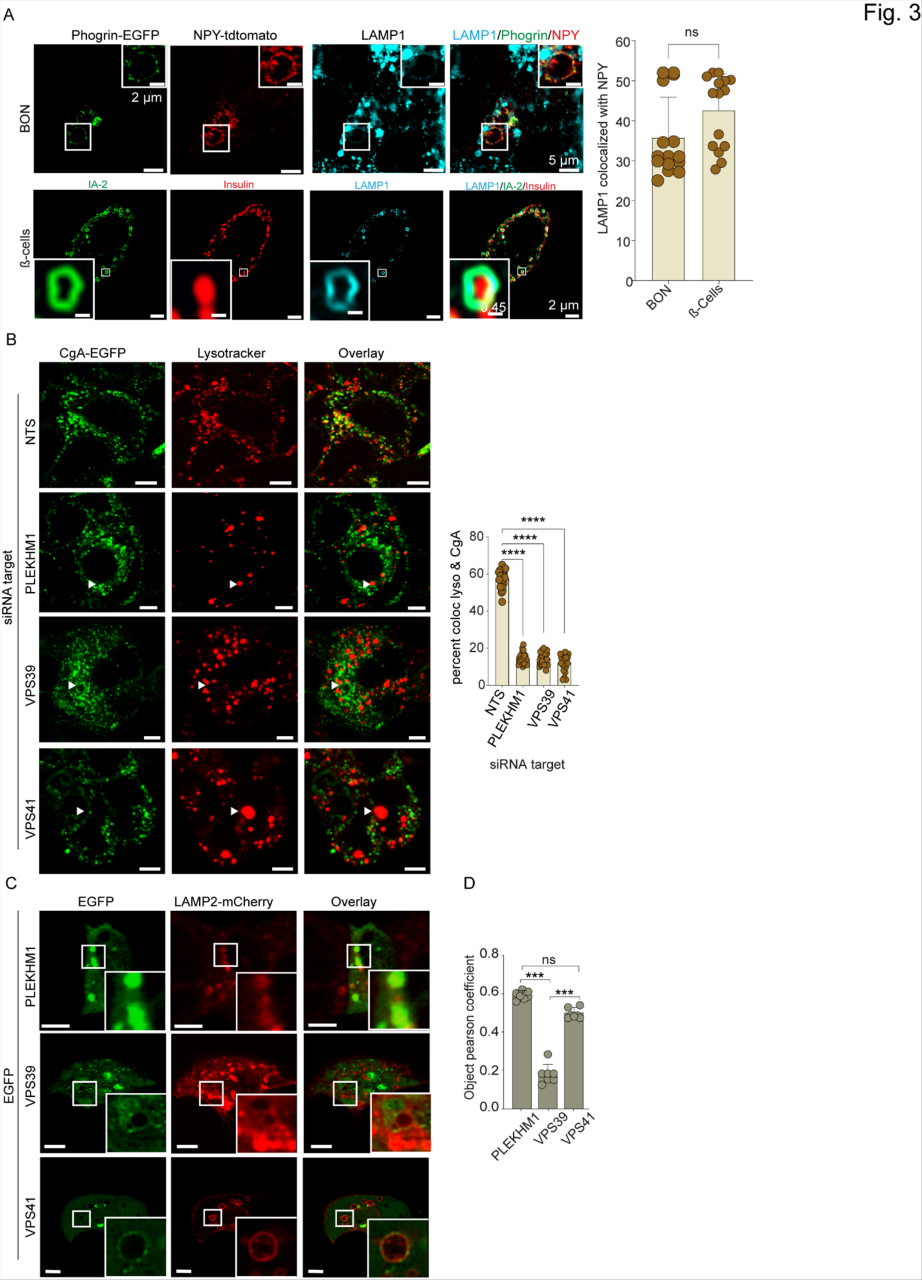
Lysosomal tethering proteins and SNAREs involved in SG-lysosome fusion. A. Upper: NPY-tomato BON cells were transfected to express Phogrin-EGFP, fixed after 16 hours of transfection, and immunostained for LAMP1. Images were acquired with a Nikon-SIM microscope. NPY-tomato was distributed in the lumen of LAMP1-positive vesicles, whereas Phogrin-EGFP was on the surface. (Scale Bar, 5 μm). Lower: Pancreatic βcells were isolated from C57N/6J mice and cultured in 5.5 mM glucose for five days. Cells were fixed and immunolabeled for IA-2, insulin, and LAMP1 with images acquired with a Nikon confocal microscope. LAMP1-positive NPY-containing organelles were counted manually and graphed as the total number per cell (n= > 15, mean ± SD, ns). (Scale Bar, 2 μm). B. CgA-GFP BON cells were treated with indicated siRNAs for 72 h. Depletion of PLEKHM1 or HOPS subunits VPS41 and VPS39 significantly impaired the colocalization of Lysotracker DND99 with CgA containing SGs. (n = 35, N = 3, mean ± SD, **** p <0.0001). (Scale Bar, 5 μm). C & D. EGFP-tagged PLEKHM1 or HOPS subunits VPS39 and VPS41 were co-expressed with LAMP2-mCherry. PLEKHM1 and VPS41 were mainly localized to LAMP2 structures. Colocalization of red puncta with green puncta per cell (n= > 15, mean ± SD, ns, ***p <0.001). (Scale Bar, 5 μm).

Previous EM studies^15,16^ and our light microscopy study (Fig. 1C) suggested that endocrine cell crinophagy involves the fusion of SGs with lysosomes. However, the mixing of SG membrane and lysosomal membrane components resulting from fusion had not previously been shown. We further characterized crinophagy in BON cells as well as in mouse pancreatic β cells by immunostaining SG membrane markers (phogrin-EGFP or IA-2b, respectively), which remained on the membrane of LAMP1-positive bodies, and SG core contents (NPY or insulin, respectively) that were internalized (Fig. 3A). The results provide evidence for membrane mixing and fusion in crinophagy. A direct SG-lysosome fusion mechanism for crinophagy underscores its unique role in mediating SG content degradation while enabling potential SG membrane protein recycling.

### Identifying lysosomal proteins involved in heterotypic SG-lysosome fusion

Heterotypic endosomal membrane fusion processes in mammalian cells such as endosome-tolysosome^32^, phagosome-to-lysosome^32^, and autophagosome-to-lysosome^33^ are facilitated by lysosomal tethering proteins PLEKHM1 and the HOPS complex. We determined whether these are required for crinophagy in neuroendocrine cells by their depletion with siRNAs. The efficient depletion of PLEKHM1 and the HOPS subunits VPS41 and VPS39 (Fig. S3A) resulted in the failure of CgA to enter lysosomes suggesting that these components are essential for crinophagy (Fig. 3B). To determine the subcellular localization of PLEKHM1, VPS39, and VPS41, we co-expressed EGFP-tagged versions with LAMP2-mCherry, a lysosomal marker (Fig. 3C& D). PLEKHM1 and VPS41 primarily colocalized with lysosomes marked by LAMP2^33^ whereas VPS39 localized to the cytoplasm or to structures lacking LAMP2 (Fig. 3D).

Membrane fusion between donor and acceptor compartments typically requires SNARE complexes^34^. We found that the depletion of STX7 and STX8 significantly reduced SG-lysosome merge (Fig. S3B). Overall, these studies indicate a critical role for lysosomal tethering proteins PLEKHM1 and HOPS subunits and lysosomal SNAREs STX7 and STX8 in mammalian neuroendocrine cell crinophagy.

### Identifying SG proteins involved in heterotypic SG-lysosome fusion

**Figure 4:**
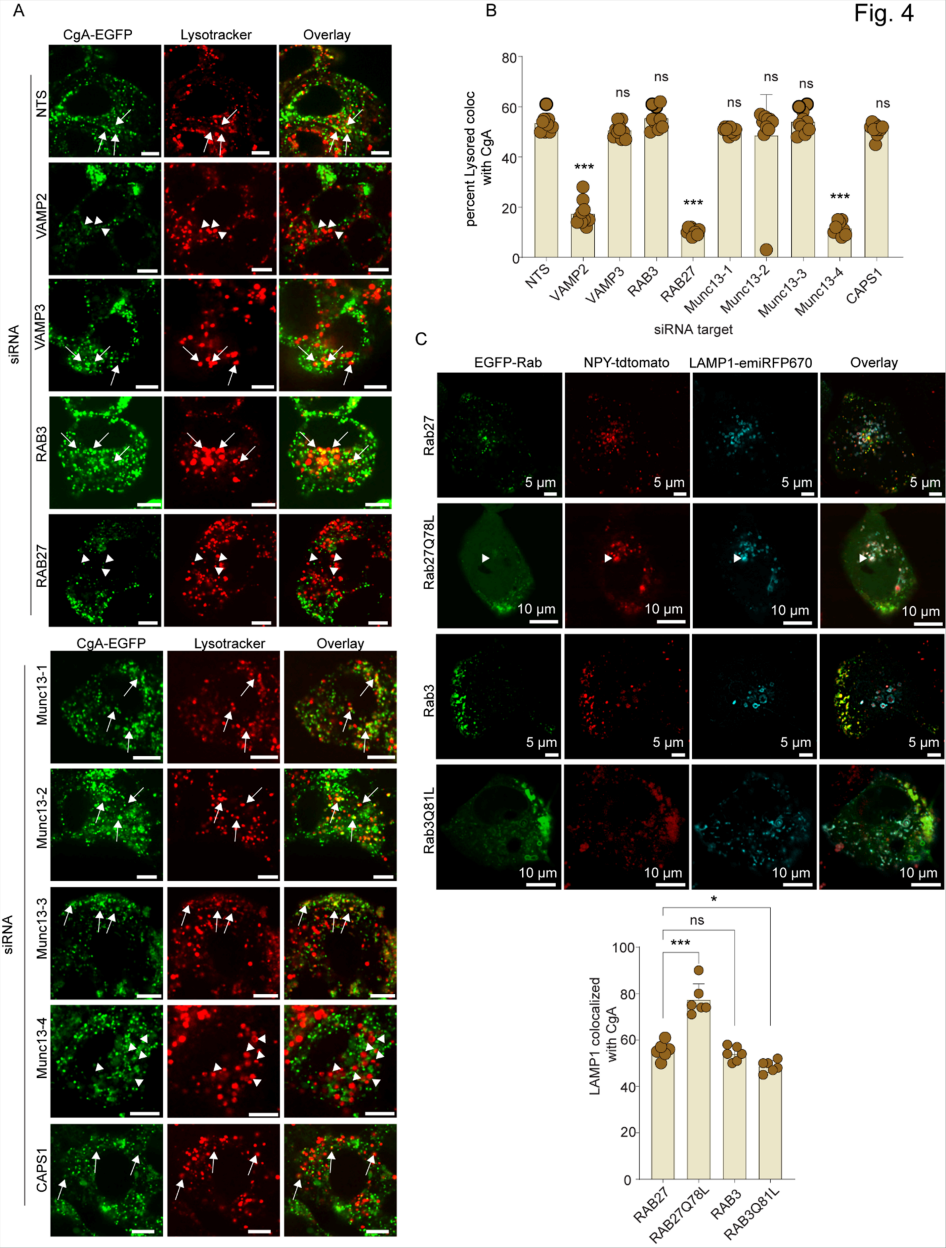
SG resident proteins and SNAREs involved in SG-lysosome merge. A & B. CgA-EGFP BON cells were treated with indicated siRNAs for 72 h. Depletion of VAMP2, RAB27, or Munc13-4 significantly impaired the colocalization of Lysotracker DND99 and CgA-containing SGs. No significant changes were observed in Munc13–1,2,3, CAPS1, VAMP3, or RAB3A knockdown. Arrows show CgA merge with lysosomes, and arrowheads show lysosomes that failed to merge with CgA. Colocalization of red puncta with green puncta per cell (n = 35, N = 3, mean ± SD, *** p <0.001, ns). (Scale Bar, 5 μm). C. Co-expression of LAMP1-emiRFP670 with EGFP tagged constitutively active RAB27A(Q78L) or RAB3A(Q81L) or wild-type Rabs was conducted in NPY-tdtomato stable BON cells. Rab27A(Q78L) expression significantly improved the targeting of NPY-containing SGs to lysosomal compartments. Arrowheads show accumulation of NPY-tdtomato inside LAMP1 labeled lysosome in cells expressing RAB27A (Q78L). Colocalization of LAMP1 with CgA per cell. (n= > 15, mean ± SD, ns, *p <0.1, ***p <0.001), (Scale Bar, 5 μm).

To identify SG protein candidates for involvement in crinophagy, we enriched SGs on StrepTactin beads (Fig. S4B) from lysates of cells expressing phogrin-EGFP-twin-strep on SGs (Fig. S4A). Western blotting identified several candidate proteins including the SNARE VAMP2, the GTPases Rab3 and Rab27A, and tethering proteins CAPS, Munc13–1, and Munc13-4 (Fig. S4C). We tested each of these proteins in the live-cell assay for SG-lysosome merge. siRNA depletion (Fig. S4D) of Rab27A, VAMP2, or Munc13-4 significantly impaired SG-lysosome merge, while knockdown of Rab3A, VAMP3, Munc13–1, Munc13–2, Munc13–3, or CAPS showed no significant impact (Fig. 4A & B). Control experiments confirmed that the reductions in SG-lysosome merge were not due to decreases in CgA-containing SG levels (Fig. S4E).

Supporting the siRNA screen results, we found that expression of a constitutively active, GTP-locked Rab27A(Q78L) mutant increased SG content entry into lysosomal compartments, whereas the GTP-locked Rab3A(Q81L) mutant did not have this effect (Fig. 4C). Overall, these studies identify Rab27A, Munc13-4, and VAMP2 on SGs as crucial for the SG-lysosome merge in crinophagy and indicate that Rab27A plays a key role.

### Munc13-4 on SGs is essential for SG degradation in neuroendocrine cells

**Figure 5:**
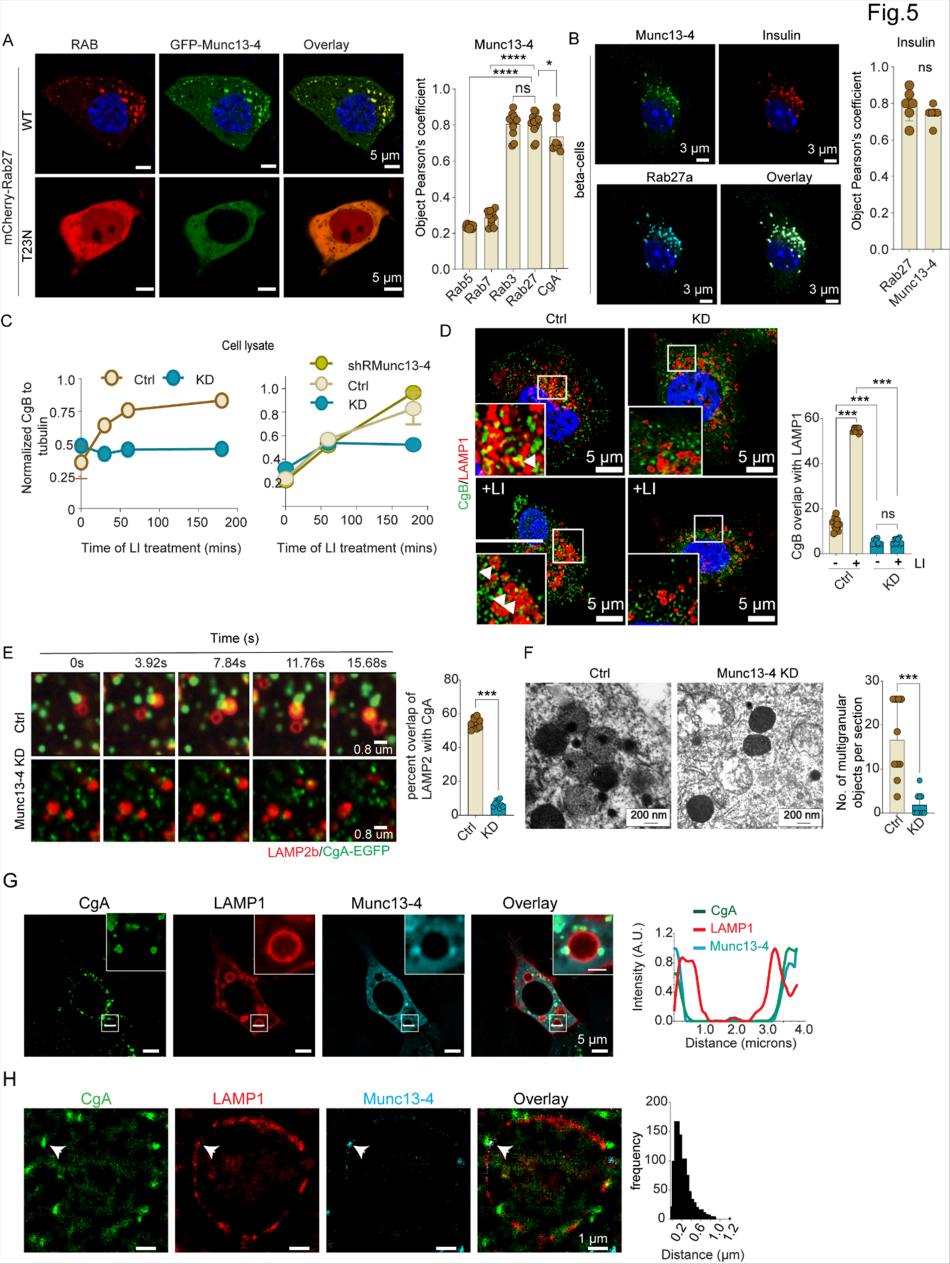
Munc13-4 localization on secretory granules and its regulatory role in SG-lysosome fusion. A. Co-expression of EGFP-Munc13-4 with mCherry-Rab27A or dominant negative -RAB27A(T32N) in BON cells. B. Immunostaining of RAB27A, Munc13-4, and insulin in mouse pancreatic β-cells. A & B- Colocalization was determined using object Pearson’s correlation coefficient. (n= > 15, mean ± SD, ns, *p <0.1, ****p <0.0001). C. BON cells (Ctrl, Munc13-4 KD, or KD cells with rescue Munc13-4 rescue) were treated with LIs for the indicated times. Cell lysates were subjected to SDS-PAGE and western blot analysis for CgB and tubulin. (means ± SD, n = 3). D. BON cells (Ctrl or Munc13-4 KD) were treated with DMSO or LIs and immunostained for CgB and LAMP1. Quantitation of overlapping CgB with LAMP (n= > 15, mean ± SD, ns, ***p <0.001). E. Live-cell imaging of CgA-EGFP and LAMP2b-mCherry in Ctrl and Munc13-4 KD cells. Quantitation of overlapping CgA-EGFP with LAMP2b-mCherry. (n = 10; N = 3 mean ± SD, ***p <0.001). F. Electron micrographs of BON cells (Ctrl and Munc13-4 KD) treated with LIs for 4 h before fixation. Multigranular objects per section were quantitated (n= 30, mean ± SD, ns, ***p <0.000). G. BON cells expressing CgA-EGFP, LAMP1-mScarlet-I, and emiRFP670-Munc13-4 were imaged live by confocal microscopy. A line graph was generated across a LAMP1-containing lysosome with bound CgA SGs. Munc13-4 was distributed to the periphery of lysosomes. H. STORM image depicting lysosome LAMP1 antibody clusters with peripheral clusters of CgB and Munc13-4 antibodies. CgB, LAMP1 & Munc13-4 antibody clusters were identified, and the histogram plotted for the distance between CgB and LAMP1 clusters. (n= > 10, N=3).

Munc13-4 is well known as a tethering factor for late endosomal membrane fusion events and the exocytosis of lysosome-related organelles in myeloid secretory cells^35–37^. Munc13-4 is ubiquitously expressed in tissues, but its function in non-myeloid cells is largely unknown with few exceptions^38,39^. A role for Munc13-4 in neuroendocrine cells has not been previously established^40^. We determined the subcellular localization of Munc13-4 in endocrine cells, which showed co-localization with CgA-containing, Rab27A-positive SGs in BON cells and with insulin-containing SGs in mouse pancreatic β cells (Fig. 5A & B). Munc13-4 was absent from Rab5- or Rab7-positive endosomes and Rab2-containing Golgi or LAMP1-positive lysosomes (Fig. S5A). The SG localization was Rab27A-dependent as Munc13-4 redistributed to the cytoplasm upon expression of a dominant-negative Rab27A mutant (Fig. 5A, lower panel). Biochemical studies confirmed Munc13-4’s presence in a phogrin-enriched, but not a LAMP1-enriched cell fraction (Fig. S5B), and a selective interaction of Munc13-4 with Rab27A over Rab3A (Fig. S5C). The results indicate that Munc13-4 resides on SGs in endocrine cells anchored by Rab27A.

To further assess the importance of Munc13-4 in SG turnover and crinophagy, we generated stable Munc13-4 knockdown (KD) BON cell lines in which Munc13-4 expression was strongly suppressed (Fig S5D & E). While Munc13-4 is essential for regulated secretion in myeloid cells^37,41,42^, its depletion in endocrine cells did not significantly affect regulated secretion (Fig. S5F). LI treatment increased CgB levels in wild-type cells as shown previously, but not in Munc13-4 knockdown cells due to the lack of CgB targeting to lysosomes (Fig. 5C & S5G). Consistent with this, lysosomes in Munc13-4 KD cells lacked CgB immunoreactivity following LI treatment (Fig. 5D). Importantly, in live-cell studies of Munc13-4 KD cells, SGs (CgA-EGFP) only transiently contacted lysosomes (LAMP2-mCherry) but SG content failed to undergo lysosomal entry (Fig. 5E, lower panels) in contrast to the entry observed in control cells (Fig. 5E, upper panels). The defect in SG turnover in Munc13-4 KD cells was fully rescued by re-expressing shRNA-resistant wild-type Munc13-4 but was not rescued by expressing a characterized Ca^2+^-binding-deficient C2A*B* Munc13-4 mutant (Fig. S5H & S5I), which has two D to N substitutions in each C2 domain to eliminate Ca^2+^ binding^43^. With the C2A*B* Munc13-4 mutant expression, SGs docked onto lysosomes but did not deliver SG content into them. We interpret these results to indicate that Munc13-4 depletion prevents docking and fusion of SGs with lysosomes, and that the C2A*B* Munc13-4 restores docking but not fusion. The latter finding suggests a Ca^2+^-dependence for fusion (also see below).

We ruled out alternative explanations for the SG turnover defect in Munc13-4 KD cells such as impaired lysosome biogenesis or endosomal trafficking. Firstly, by using Magic Red to monitor intracellular cathepsin protease activity, we showed that lysosomal activity was comparable in Munc13-4 KD and control cells. Secondly, DQ-BSA was properly targeted to and de-quenched in lysosomes in both cell types (Fig S5K). However, while CgA-EGFP-containing SGs appropriately colocalized with lysosomal DQ-BSA in control cells, SGs failed to colocalize with lysosomes in Munc13-4 KD cells. The results indicate that lysosomal function was normal in Munc13-4 KD cells but SG targeting to lysosomes was specifically impaired.

Multigranular bodies in endocrine cells detected by EM were considered to be generated from a lysosomal merge with SGs^15,22,44^. We found a marked reduction in the number of multigranular bodies by EM in Munc13-4 KD BON cells compared to control cells (Fig. 5F). Similarly, there were fewer multigranular bodies and more intact insulin SGs in pancreatic b cells isolated from Munc13-4 KO mice (Fig S5J). The results were consistent with a role for Munc13-4 in targeting SGs into a degradative lysosomal compartment in BON and mouse pancreatic b cells.

The preceding results indicating that Munc13-4 is critical factor for docking and fusion suggest that Munc13-4 functions at the SG-lysosome interface. We conducted 3-channel confocal fluorescence studies to localize Munc13-4 on CgA-containing SGs during contact with LAMP1-containing lysosomes. Overexpressing emiRFP670-Munc13-4 increased the number of SGs docked on LAMP1-mScarlet-labeled lysosomes, and a scan suggested that Munc13-4 was positioned between CgA-EGFP-containing SGs and the lysosomal LAMP1-containing surface at low resolution (Fig. 5G). At higher resolution in STORM microscopy with LI treatment to increase lysosome size (Fig. 5H), SGs as CgA-containing clusters were in proximity (~ 0.2μm) to the lysosomal LAMP1-containing surface at a site (arrowhead) where an SG docked onto lysosomes. CgA was also internal in the lysosomes indicating that fusion with SG content insertion had occurred. Munc13-4 clusters were on the LAMP1 surface close to CgA clusters. The results suggest that Munc13-4 is positioned at the interface where SGs dock and fuse onto lysosomes.

### Munc13-4 interacts with Rab, SNARE, and tether proteins

Membrane fusion reactions are catalyzed by SNARE protein complexes assembled at the interface of membrane compartments by tethering proteins commonly anchored to a membrane compartment by Rab proteins^34^. Guided by our results on proteins functionally important for crinophagy, we performed targeted screens to identify potential Munc13-4 interaction partners.

**Figure 6:**
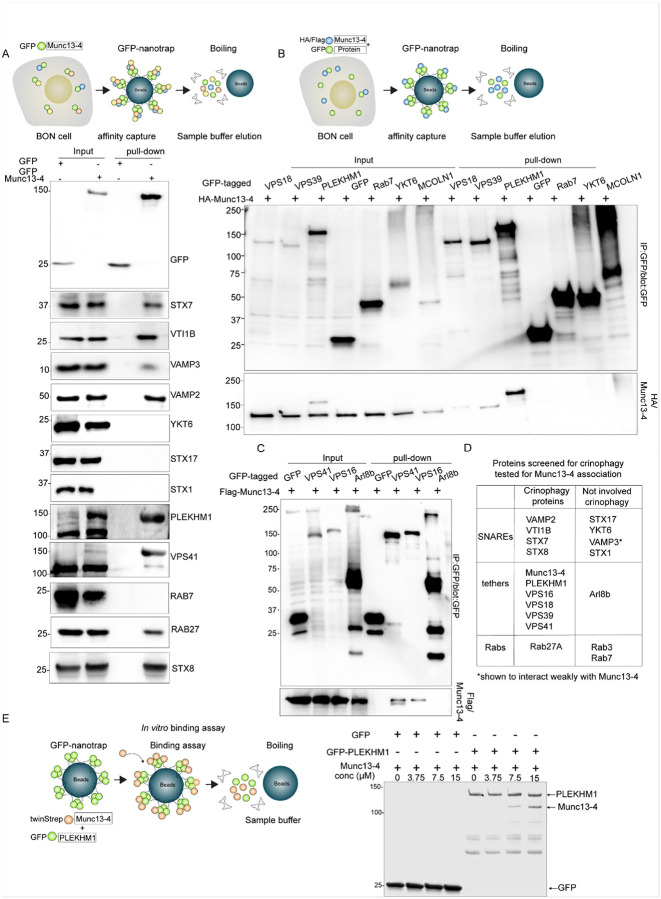
Munc13-4 interacts with tethers and SNAREs. A. Lysates of BON cells expressing GFP or GFP-Munc13-4 were immunoprecipitated with GFP nanobodies. Pull-downs were analyzed by western blotting with the indicated antibodies. B. Lysates of BON cells co-expressing GFP-tagged VPS18, VPS39, PLEKHM1, Rab7, YKT6, MCOLN1, and HA-tagged Munc13-4 were immunoprecipitated with GFP nanobodies. Pull-downs were subjected to western blotting with HA or GFP antibodies. C. Lysates of BON cells co-expressing GFP-tagged VPS41, VPS16, ARL8B, and Flag-Munc13-4 were immunoprecipitated with GFP nanobodies. Pull-downs were western blotted with HA or Flag antibodies. D. Proteins that interact with Munc13-4 correspond to those essential for crinophagy (column 1). Proteins not essential for crinophagy did not detectably interact with Munc13-4 (column 2). E. Immobilized GFP or GFP-PLEKHM1 was incubated with the indicated concentrations of Munc13-4 followed by SDS-PAGE and staining with Imperial blue.

In the first screen, we expressed GFP-tagged Munc13-4 in BON cells and performed pulldowns using a GFP nanobody (Fig. 6A). Immunoblotting revealed Munc13-4 association with the SG-resident SNARE VAMP2, and with endosomal SNAREs STX7, STX8, and VTI1B. In contrast, we did not detect interactions with other SNAREs such as VAMP3, STX1, STX17, or YKT6. As expected, GFP-Munc13-4 also coprecipitated with Rab27A but not with Rab7. In addition, we observed Munc13-4 association with the tethering factor PLEKHM1 and the HOPS complex subunit VPS41.

In a second screen, we co-expressed candidate proteins as GFP fusions along with HA- or FLAG-tagged Munc13-4 to further validate associations (Fig. 6B & C). This approach confirmed Munc13-4 interactions with additional HOPS subunits (VPS16, VPS18, and VPS39) as well as with PLEKHM1. Munc13-4 associations with Rab7, YKT6, MCOLN1, Arl8b, or ESCRT-I complex proteins were not detected (Fig. S6A). These findings show that Munc13-4 associates with key components of the SG-lysosome fusion machinery, including SNAREs, Rab27A, PLEKHM1, and the HOPS tethering complex (Fig. 6D), supporting a role as a central regulator of crinophagy.

These studies did not determine whether protein associations with Munc13-4 were direct or indirect. Direct Munc13-4 interactions with Rab27A^37,45^ and with late endosomal SNARE proteins^35,46^ have previously been reported. Because the pulldown screens consistently found PLEKHM1 to be a Munc13-4 interaction partner, we tested for direct binding *in vitro* with purified proteins (Fig. 6E) and found that Munc13-4 binds immobilized PLEKHM1 over a micromolar concentration range (Fig. 6D). Thus, Munc13-4 can directly bind to PLEKHM1, a scaffolding protein on lysosomes that functions in endocytic, autophagic, and phagocytic vesicle interactions with lysosomes^47^. The Munc13-4 association with multiple HOPS complex subunits (Fig. 6A–C) could be mediated indirectly by binding to PLEKHM1, which binds to HOPS subunits^33^. Because Munc13-4 directly interacts with the lysosomal protein PLEKHM1, we suggest that a complex with Munc13-4, PLEKHM1, and HOPS could be responsible for tethering SGs to lysosomes.

### Munc13-4 with Ca stimulates SG-lysosome docking /fusion

**Figure 7:**
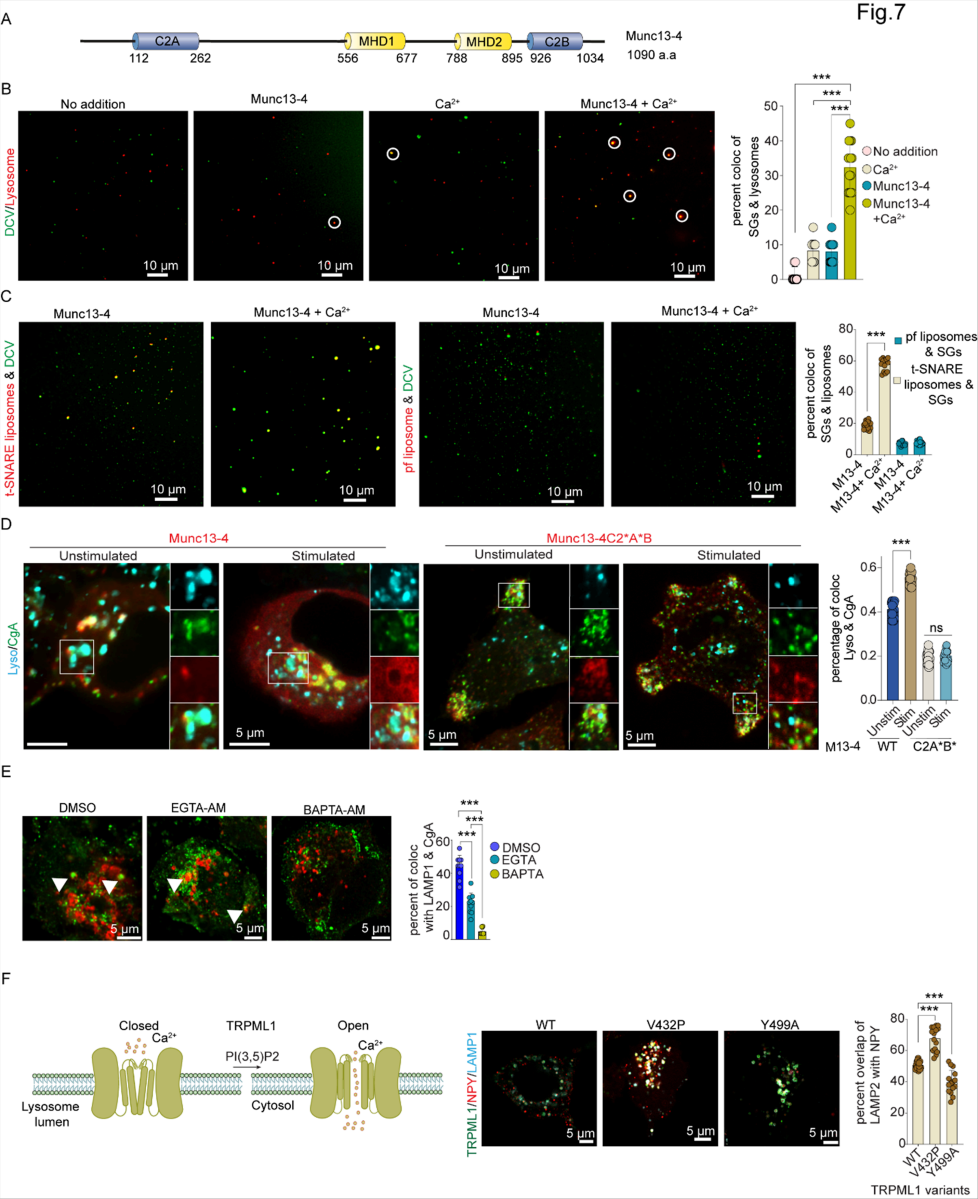
Munc13-4 mediated heterotypic fusion is Ca^2+^-regulated. A. A schematic of Munc13-4 sequence showing N- and C-terminal C2 domains flanking Munc13 homology domains. B. Phogrin-EGFP-twin-strep-tagged SGs (green channel) and LAMP1-mScarlet-I tagged lysosomes (red channel) were incubated with or without Munc13-4 and Ca^2+^ as indicated. These are shown as representative micrographs of docking/fusion reactions following immobilization on glass coverslips. Docked or fused organelles were identified by colocalization (yellow) and quantified across >500 objects per image (n = 20; mean ± SD, ***p <0.001). C. Left panels: Phogrin-enriched SGs (green) and STX7/STX8/VTI1B-containing liposomes (red) were incubated with Munc13-4 with or without Ca^2+^ as indicated. Right panels: Phogrin-enriched SGs (green) and protein-free liposomes (red) were incubated with Munc13-4 with or without Ca^2+^ as indicated. Representative micrographs of docking/fusion reactions are shown following immobilization on glass coverslips. Docked/fused organelles were identified by colocalization (yellow) and quantitated across >500 objects per image. (n = 20; mean ± SD, ***p <0.001). D. BON cells expressing mCherry-Munc13-4 or -Munc13-4 C2A*B* and CgA-EGFP were labeled with lysoblue. Indicated cells were stimulated with carbachol for 1 minute and imaged live using a confocal microscope. Inset images show Munc13-4 localized with enlarged vesicles (red) containing CgA (green) and lysoblue (cyan), which were absent in cells expressing Munc13-4 C2A*B*. Colocalization of lysoblue with CgA-EGFP was quantified. (n = 10; mean ± SD, ***p <0.001). E. BON cells were treated with DMSO, EGTA-AM, or BAPTA-AM for 30 minutes followed by LI incubation. Fixed cells were stained for CgB and LAMP1 with overlapping objects quantitated (arrowhead) (n = 15; mean ± SD, ***p <0.001). F. CgA-GFP stable BON cells co-expressing GFP-tagged WT TRPML1 or V432P or Y499A TRPML variants and LAMP1-emiRFP670 were treated with LI for 3 hours. Quantitation of CgB with LAMP1 overlap was plotted (n = 15; mean ± SD, ***p <0.001).

Munc13-4 contains a Munc13 homology domain that in Munc13–1 binds SNARE proteins and is flanked by two Ca^2+^-binding C2 domains C2A and C2B^48,49^. Indeed, Munc13-4 is Ca^2+^-dependent in binding to SNARE proteins, in stimulating SNARE-dependent liposome fusion, and in regulating membrane fusion^43,50^. However, Ca^2+^ binding to Munc13-4 is not required for its SG localization as indicated by the normal SG localization of the Ca^2+^-binding-deficient C2A*B* Munc13-4 mutant (Fig S7A & B). We assessed the Ca^2+^ dependence of Munc13-4 in an assay mimicking crinophagy by incubating SGs with lysosomes *in vitro*. SGs and lysosomes were prepared from cells expressing phogrin-EGFP-twin-strep or LAMP1-mScarlet-twin-strep, respectively (Figs S4B,C and S7A). Incubating SGs with lysosomes without Ca^2+^ at 37°C for 15–30 min failed to merge the organelles. Addition of Munc13-4 or Ca^2+^ alone did not increase the merge whereas Munc13-4 plus Ca^2+^ addition promoted a significant > 3-fold increase in the merge of organelles (Fig. 7B). Some of the merged organelles increased in size and displayed both phogrin-EGFP and LAMP1-mScarlet-I in the limiting membrane suggesting that fusion occurred. We also detected a Ca^2+^-dependent merge of VAMP2-containing SGs with STX7/STX8/VTI1B-containing liposomes in the presence of Munc13-4 (Fig. 7C, left two panels) that was dependent on SNARE proteins in the liposomes (Fig. 7C, right two panels). These results indicate that Munc13-4 promotes the Ca^2+^-dependent docking/fusion of donor and acceptor membranes containing the SNARE proteins identified to mediate crinophagy. Based on previous studies^43,50^, we attribute the Ca^2+^ dependence of these reactions to the C2 domains in Munc13-4.

### Crinophagy is Ca-regulated in endocrine cells

To investigate the role of Ca^2+^ in crinophagy, we examined SG content delivery to lysosomes under various conditions. Carbachol, a muscarinic receptor agonist that mobilizes intracellular Ca^2+^ stores in BON cells^51^, significantly enhanced crinophagy as shown by increased co-localization of SG and lysosomal markers (Fig. 7D). The increased co-localization was blocked by overexpressing the Ca^2+^-binding-deficient Munc13-4 C2A*B* mutant, suggesting that Ca^2+^ binding to Munc13-4 is essential for crinophagy.

We further tested the role of Ca^2+^ under basal conditions using cell-permeant chelators. BAPTA-AM, a fast-binding chelator, markedly reduced CgB-LAMP1 co-localization, while EGTA-AM was less effective (Fig. 7E). CgB degradation assays supported these findings (Fig. S7D) reinforcing that Ca^2+^ -activates crinophagy.

The stronger inhibition by BAPTA-AM suggested a possible role for localized Ca^2+^ flux. TRPML1, a lysosomal cation channel was suggested to play a role in in phagosome-lysosome fusion^52^. Expression of the constitutively active TRPML1(V432P) channel^53^ enhanced NPY-LAMP1 colocalization by immunostaining (Fig. 7F) and increased CgB levels after 180 min of LI-treatment (Fig. S7E). In contrast, the Ca^2+^-release-deficient TRPML1(Y499A) mutant^54^ reduced NPY-LAMP1 colocalization (Fig. 7F) and failed to increase CgB levels in the presence of lysosomal inhibitors (Fig. S7E) indicating impaired lysosomal degradation. TRPML1 activity depends on the lysosomal lipid PI(3,5)P₂^53,55,56^, and we found that expression of kinase-dead PIKfyve(K1831E), which reduces PI(3,5)P₂ levels, impaired the increase CgB levels (Fig. S7F). Similarly, siRNA knockdown of TRPML1 or PIKfyve markedly reduced SG content entry into lysosomes (Fig. S7G). Together, these results suggest that localized Ca^2+^ release via TRPML1 activates Munc13-4 to facilitate SG cargo delivery to lysosomes during crinophagy.

## Discussion

The degradation of SGs in mammalian endocrine cells remains poorly characterized at the molecular level despite critical roles in endocrine regulation^12,57^ and disease processes^8,10,58–60^. Electron microscopy studies provided a morphological framework for SG degradation in pituitary and pancreatic cells showing that SGs merge with lysosomes to form multigranular bodies in a process termed crinophagy^15,16,44^. However, the molecular mechanisms governing crinophagy in mammalian endocrine cells remained largely undefined.

Our findings show that crinophagy is the primary mechanism for SG content turnover in unstressed mammalian endocrine cells. Live-cell microscopy showed that SG-lysosome interactions transition rapidly (~ 15s) into a merged structure, likely a multigranular body, where SG contents are inserted into the lumen whereas SG membrane proteins mix with LAMP1 in the membrane providing additional evidence that crinophagy occurs *via* direct membrane fusion. Notably, in unstressed cultured endocrine cells, we observed no SG turnover through macroautophagy or microautophagy contrasting with previous studies of SG degradation under metabolic stress or gene editing conditions^8–10,60^. We showed that crinophagy operates independently of the ATG, tether and SNARE proteins required for macroautophagy^61^ and the ATG and ESCRT proteins required for microautophagy^13^. Unlike macro- and microautophagy, crinophagy selectively degrades SG contents while preserving SG membrane components for recycling.

Our findings agree with previous observations indicating the preferential degradation of older SGs^7^. The specific mechanisms that distinguish aged SGs for lysosomal degradation remain unclear and may involve cytoskeletal trafficking pathways. Recent proteomic studies identified RAB3 and KIF5b as markers for newly synthesized SGs that preferentially undergo exocytosis rather than degradation^62^. SGs in endocrine cells contain both RAB3A and RAB27A and our data indicate that RAB27A but not RAB3A is crucial for crinophagy. A constitutively active RAB27A promoted crinophagy, whereas a constitutively active RAB3A did not, suggesting that an increased RAB27 to RAB3 activity ratio during SG aging may facilitate SG targeting to lysosomes. This idea is supported by the increased crinophagy observed in pancreatic cells from RAB3A knockout mice^8^. Targeting aged SGs for lysosomal degradation may be important for removing damaged peptide hormones particularly during excessive granule production or impaired secretion.

Using a live cell assay for crinophagy with siRNA screening, we identified the key SG and lysosomal components required for crinophagy as RAB27A, Munc13-4, and VAMP2 on SGs, and STX7, STX8, VTI1B, HOPS subunits, and PLEKHM1 on lysosomes. A central finding is the identification of Munc13-4 as a major tethering/priming factor for endocrine crinophagy. Munc13-4 localizes to SGs by binding RAB27A, and live-cell imaging confirmed the necessity for both RAB27A and Munc13-4 for SG-lysosome fusion. Munc13-4 selectively interacted with RAB27A but not with RAB7 or RAB3 in protein interaction screens. We found that Munc13-4 plays multiple roles in facilitating SG-lysosome docking and fusion as described below. Munc13-4 knockdown in BON cells significantly reduced SG-lysosome co-localization and impaired SG CgB content turnover. Re-expression of active Munc13-4 in Munc13-4 KD cells restored crinophagy, whereas inactive Munc13-4 mutants failed to. Munc13-4 knockout mice exhibited fewer multigranular bodies and a greater number of insulin-containing SGs in pancreatic b cells indicating reduced crinophagic flux. The results highlight Munc13-4 as a critical regulator of SG homeostasis in peptidergic endocrine cells.

SG-lysosome fusion requires SNARE proteins. The SNAREs functionally important for crinophagy were identified in siRNA screening as VAMP2, STX7, and STX8. Munc13-4 was found to associate with late endosomal SNAREs STX7, STX8, and VTI1B in pull-down assays as well as with the SG-localized SNARE VAMP2. Moreover, Munc13-4 was able to promote docking/fusion on donor and acceptor liposomes containing these SNARE proteins. This putative SNARE complex VAMP2/STX7/STX8/VTI1B identified for mammalian crinophagy has not been observed in other fusion pathways but does form *in vitro* and has been detected in cells^63,64^. These studies provide a distinct Munc13-4-dependent fusion pathway for crinophagy in mammalian endocrine cells that uses SNARE complexes that differ from the VAMP8/STX17/SNAP29^65^ and STX7/SNAP29/YKT6^65^ complexes that mediate autophagosome-lysosome fusion; from VAMP7/STX7/STX8/VTI1B complexes that mediate late endosome-lysosome fusion^66^; from VAMP7/VAMP8/STX7/SNAP23 complexes that mediate phagosome-lysosome fusion^67^; and from STX13/SNAP29/YKT6 or VAMP7 complexes that mediate *Drosophila* glue granule-lysosome fusion^18,19^.

Our findings are consistent with recent studies on the degradation of proinsulin-containing iSGs in pancreatic β cells reporting that Golgi-derived VAMP4 on iSGs interacts with lysosomal SNAREs STX7, STX8, and VTI1B to mediate crinophagy in metabolic stress^68,69^. VAMP4 is removed during iSG conversion to mature SGs where VAMP2 is the dominant isoform. This suggests that mature SGs under unstressed conditions would be degraded by the constitutive VAMP2/STX7/STX8/VTI1B pathway identified here. Further studies are needed to determine whether this metabolic checkpoint for iSG degradation is restricted to pancreatic b cells or is a conserved feature across peptidergic endocrine cells. We found Munc13-4 is present on the total population of insulin SGs in pancreatic β cells using an antibody that detects both insulin and proinsulin, which raises the possibility that Munc13-4 may also function in the stress-induced turnover of iSGs reported for pancreatic b cells^68,69^.

Membrane fusion commonly requires sets of tethering factors that assemble and stabilize SNARE complexes^70^. Crinophagy requires HOPS and PLEKHM1 complexes, which are also important for other lysosomal fusion events^18,63,65,67,71^. In protein interaction screens, Munc13-4 associated with PLEKHM1 and HOPS complex subunits VPS16, VPS18, VPS39, and VPS41 possibly indicating that Munc13-4 on SGs acts to bridge SGs to lysosomes in tethering/docking interactions but this will need to be directly tested. The HOPS complex and PLEKHM1 interact with each other and with other fusion-related proteins^47^ but we did not detect other reported HOPS- or PLEKHM1-interacting proteins (e.g., BIRC6, EPG5, Arl8b, Rab7, GRASP55) in our screens. A limitation of our approach was that we screened candidate proteins known from other fusion pathways indicating we may have missed other essential proteins or proteins that exhibit redundancy. The protein interaction studies indicated that Munc13-4 associates with all the proteins that were identified as essential for crinophagy (Fig. 6D) whereas many other Rab, SNARE, or tethering factors were not detected in the Munc13-4 interaction studies.

Munc13-4 has distinguishable roles in crinophagy for SG-lysosome docking and fusion steps. Overexpression of Munc13-4 in BON cells significantly increased SG-lysosome associations, while Munc13-4 depletion reduced them. Rescue studies in Munc13-4 KD cells indicated that the Ca^2+^-binding-deficient Munc13-4 mutant C2A*B* arrested SG-lysosome interactions at the docking stage preventing fusion. High resolution STORM studies localized Munc13-4 to the SG-lysosome interface consistent with a role in SG docking. However, Ca^2+^ binding to Munc13-4 is necessary for a post-docking step that leads to fusion. Our *in vitro* studies showed that Munc13-4 enhances Ca^2+^-dependent docking/fusion of SGs with lysosomes. Previous studies showed that Munc13-4 interacts with membrane phospholipids and SNARE proteins in a Ca^2+^-stimulated manner consistent with Munc13-4 mediating a Ca^2+^-dependent fusion step^43,50^.

A key finding of this study is that crinophagy in endocrine cells is regulated by Ca^2+^. This was suggested by the inhibition of carbachol-stimulated crinophagy by overexpression of the Ca^2+^-binding deficient Munc13-4 C2A*B* mutant. In addition, the Ca^2+^ chelators BAPTA and EGTA were found to inhibit SG-lysosome fusion. The stronger effect of BAPTA suggests that a local source of lysosomal Ca^2+^ may regulate crinophagy similarly to other lysosomal fusion events^72,73^. The activation of TRPML1, a lysosomal cation channel may contribute to this local Ca^2+^ source as shown by the expression of an active TRPML1 form that enhanced crinophagy while an unregulated Y99A mutant reduced it. The inhibition of SG-lysosome fusion by a dominant-negative PIKfyve mutant aligns with evidence that phosphatidylinositol 3,5-bisphosphate activates TRPML1^55^. Thus, Munc13-4 plays a role in docking SGs on lysosomes and at a Ca^2+^-dependent post-docking fusion event.

There is considerable interest in lysosomal fusion events for the proteins that respond to local Ca^2+^ levels. These are proposed to be synaptotagmin 7 for lysosome-plasma membrane fusion^74^, calmodulin for endosome-lysosome fusion^75,76^, and ALG-2 for autophagosome-lysosome fusion^77^. Based on our findings that Munc13-4 is on SGs, that SGs dock onto lysosomes via Munc13-4, that Ca^2+^ binding to Munc13-4 is required for SG-lysosome fusion, and that Munc13-4 localizes to the SG-lysosome interface, Munc13-4 is likely the Ca^2+^ sensor poised near local Ca^2+^ increases at the interface of SGs with lysosomes with a role to drive SNARE complex formation for fusion in crinophagy^43,50^.

Crinophagy is essential for maintaining endocrine cell homeostasis by degrading aged or unused SGs that may harbor dysfunctional peptides, or secretion-incompetent SGs that are dysfunctional^8,22^. Crinophagy occurs continuously in resting cells where it is responsive to resting cytoplasmic Ca^2+^ levels. We show that crinophagy is also accelerated by increased Ca^2+^ levels that would also stimulate the exocytosis of newer primed SGs^6,78^. Thus, the Ca^2+^-dependent enhancement of crinophagy may serve the dual purpose of facilitating the degradation of unused SG content as well as the recycling of SG membrane components via the Golgi for the packaging of new peptide hormones, overall promoting SG turnover. It will be important to determine how SG aging is signaled and whether any of the components identified here such as Rab27A and its effector Munc13-4 are modified during SG aging to play a role in directing older SGs to lysosomes.

## Materials and Methods

### Cell lines and cell culture

BON cells were established from a pancreatic carcinoid and provided by Dr. James C. Thompson (University of Texas, Galveston, TX, USA). Cells were maintained in media composed of DMEM (Gibco #11965–092)/Ham’s F12 (Gibco # 11765054) (1:1) with 10% FBS (Gibco # 1600–044) at 37°C with 5% CO_2_. Cells were maintained in antibiotic-free medium and dissociated for subculture using TrypLE Express (Thermo Fisher Scientific #12605–010). All cell lines in this study were not used beyond passage 30 from the original derivation and were routinely tested for mycoplasma contamination.

### Generation of stable cell line using lentivirus

DNA encoding mouse CgA-EGFP from Ricardo Borges (University of La Laguna, Spain) was subcloned into the pLVX lentiviral vector using HifiAssembly (NEB #E2621S). HEK 293FT cells (Invitrogen) produced the CgA-EGFP virus by transfecting with envelope pMD2.G (Addgene #12259) and packaging plasmid psPAX2 (Addgene #12260) via Lipofectamine 2000 (Thermo Fisher #11668019). After 60 hours of transfection, the media was filtered using a 0.45 μm sterile syringe filter (VMR #28145–481), and viral particles were collected by ultracentrifugation at 33,000 rpm for 2 hours. The concentrated virus was resuspended in 100 μl of ice-cold PBS and used to transduce BON cells with 10 μg/ml protamine sulfate (Sigma #4020). Clones were selected and tested for exocytic activity and relative fluorescent expression.

A similar approach was used in generating Munc13-4 KD knockdown BON cells. shRNA sequence targeted human Munc13-4 5−CATCAGCGGTGGATCTATC − 3 targeting 1883–1901 of human Munc13-4 mRNA. A non-targeting lentivirus was used as a control. In rescue experiments we used shRNA-resistant Munc13-4 (vector builder pLV[Exp]-Bsd-CMV > Myc/hUNC13D [NM_19924 2.2]) Two sets of site-directed mutagenesis using QuikChange XL(Agilent #200516) carried out using set 1 forward 5−*GA*ℂ∀*GCACAGCACTCTGCAGTGGATCTAT*ℂ*A*ℂ − 3 set 1 reverse 5 – *GGTGGATAGAT*ℂ*ACTGCAGAGGTGCTGTGC*Τ*GGTC*−3and set 2 forward 5 – *CAGCA*ℂ*TCTGCAGTAGAC*Τ*AT*ℂ*A*ℂ*CTGC* Τ *TG* − 3and set 2 reverse 5 *C*∀*AGCAGGTGGAT*∀*GTCTACTGCAGAGGTGCTG* − 3.

A similar method was used to create NPY-tdtomato-stable BON cells. NPY and tdtomato constructs were subcloned into a pLVX vector using Hifi Assembly. Cells were plated in low density, selected with a microscope, and transferred to 24-well dishes. Clones were evaluated for exocytic activity and fluorescence. For stable LAMP1-mScarlet-I BON cells, LAMP1-mScarlet-I was transfected using lipofectamine 3000 (Thermo Fisher Scientific #L3000001). Cells were plated in low density, selected microscopically, and transferred to 24-well dishes, with clones assessed for exocytic activity and fluorescence.

NPY-HaloTag stable BON cells were generated by transfecting a plasmid encoding NPY-HaloTag using Lipofectamine^™^ 3000 Transfection Reagent (#L3000008, Invitrogen^™^). Transfected BON cells were labeled with Janelia Fluor^®^ 646 HaloTag^®^ Ligand (#HT1060, Promega). NPY-HaloTag positive cells were sorted using a BD FACSDiscover S8 at the UWCCC (University of Wisconsin Carbone Cancer Center) into 96-well plates as single cells, and clones were expanded for further analysis.

### Immunostaining

For immunostaining studies, cells were briefly washed with serum-free DMEM, and the media was aspirated. To visualize the SG content marker chromogranin A (CgA) within lysosomes, cells were treated with a lysosomal inhibitor cocktail consisting of 1 μM Pepstatin A (Sigma-Aldrich, #P5318) and 1 μM E64D (Cayman Chemical, #13533) in DMEM containing 1% FBS. This treatment was carried out for 3–4 hours at 37°C to prevent lysosomal degradation of CgA and preserve its epitopes for antibody recognition.

Following treatment, cells were fixed with 4% paraformaldehyde (Polysciences, #18814) for 15 minutes at room temperature and washed three times with PBS. Residual aldehydes were quenched with 0.25% glycine in PBS. Cells were then immunostained using a primary antibody against LAMP1 to label lysosomes and a primary antibody against CgA to label secretory granules. Nuclei were counterstained with DAPI. Images were acquired using a Nikon A1R + confocal microscope. Colocalization between LAMP1 and CgA was quantified using image analysis software. Data were analyzed to determine the degree of overlap between lysosomal and SG markers, and statistical significance was assessed as indicated.

### Timer imaging

For timer studies, BON cells were transfected with a plasmid encoding chromogranin A fused to the fluorescent timer protein (CgA-MediumFT). The MediumFT reporter enables differentiation between newly synthesized and aged granules based on chromophore maturation, with blue fluorescence (excitation/emission: ~390/460 nm) indicating newly synthesized protein and red fluorescence (~ 570/615 nm) indicating aged protein. Imaging was performed approximately 3.9 hours post-transfection to capture the dynamics of vesicle maturation. Cells were imaged live using a Nikon A1R + confocal microscope with appropriate filter sets to distinguish blue, red, and green channels. For colocalization analysis, fluorescence intensities in regions of interest (ROIs) were quantified, and background fluorescence was subtracted using ROIs from nonfluorescent cells on the same coverslip. Colocalization of new (blue) and old (red) CgA granules with LAMP2-positive compartments was analyzed and expressed as the percentage of overlap.

### Lysosomal inhibitor treatment and western blot analysis

BON cells were cultured in complete DMEM and treated with a lysosomal inhibitor (LI) cocktail consisting of 1 μM Pepstatin A (Sigma-Aldrich, #P5318) and 1 μM E64D (Cayman Chemical, #13533) for 0, 30, 60, or 180 minutes at 37°C. For control conditions, an equivalent volume of (i.e., 0.1%) DMSO was added. For analysis of total cell lysates, cells were collected at each time point, lysed in lysis buffer supplemented with protease inhibitors, and total protein concentration was determined. SDS-PAGE separated equal amounts of protein, transferred to nitrocellulose membranes, and immunoblotted using antibodies against chromogranin B (CgB) and β-actin, which served as a loading control. Densitometric analysis of CgB bands was performed using ImageJ, and CgB signal was normalized to β-actin. Data represent mean ± SD from three independent experiments. For knockdown or rescue experiments, the same protocol was followed following transient expression of the protein of interest.

For secreted protein analysis, culture media were collected at each time point from untreated (or DMSO-treated) and LI-treated cells. The media were cleared of cell debris by centrifugation and then subjected to methanol precipitation. The resulting protein pellets were resuspended in Laemmli sample buffer, resolved by SDS-PAGE, and immunoblotted for CgB. Densitometric analysis of CgB bands was conducted using ImageJ, and data represent mean ± SD from three biological replicates.

### RNA interference and transfection

All siRNA pools used in this study were purchased directly from Dharmacon or obtained from the Small Molecule Screening Facility (SMSF) at The University of Wisconsin Carbone Cancer Center. siRNAs were delivered into BON cells by reverse transfection. In brief, siRNA (50 nM final) was mixed with RNAiMAX (Thermo Fisher Scientific # 13778150) reagent in OptiMEM (Thermo Fisher Scientific # 31985070) and transfection buffer in 96-well plate wells, and the cell suspension was added (1.8 × 10^4^ cells per well) into poly-D-lysine coated (Sigma # P6407) μ-Slide 8 Well Glass Bottom (ibidi # 80827) or FluoroDish (WPI #FD35PDL-100). At 72 h before imaging, cells were treated 5 nM LysoTracker Red DND-99 (Thermo Fisher Scientific #L7528) or 5 nM LysoView 594 (Biotium # #70084) for 15 minutes. Cells were imaged 72 h after transfection using Nikon Yokogawa W1 CSU Spinning Disk Confocal Microscope or a Nikon A1R + confocal system.

For colocalization analysis, 25–30 cells per condition were analyzed per experiment, across three independent replicates. To quantify crinophagy, lysosomes (LysoTracker-positive vesicles) and SGs (CgA-EGFP–positive vesicles) were segmented as discrete 3D objects using Imaris Bitplane software (version 9.9). Images were background-subtracted, and fluorescent objects were identified using the Spot module in Imaris (Bitplane). Colocalization was quantified by calculating the percentage of objects overlapping within a 250 nm distance. This method calculates the degree of overlap between CgA-EGFP and lysosomal markers, representing the proportion of SGs undergoing crinophagy. Additionally, the SVI Huygens (version 24) Colocalization plugin was used to compute overlap coefficients based on intersecting volumes between red (lysosomal) and green (CgA-EGFP) objects. All image analysis was performed in a blinded manner, and thresholding parameters were applied uniformly across all experimental conditions to minimize bias.

### Secretion assay

BON cells were seeded 18 hours before media collection to reach 70–80% confluence. Cells were washed thrice in PBS + 100 μM EDTA before media collection. Stimulation with 1.25 μM ionomycin (Sigma #10634) was limited to 20 min in the presence of regular media. Spent media was centrifuged at 300 g for 5 min, at 1,000 g for 10 min to remove cellular materials, and at 10,000 g for 10 min to provide supernatants for filtering onto nitrocellulose membrane using a Slot-Blot apparatus (Schleicher & Schuell). Total cellular material was acquired and loaded on the membrane to allow for the quantification of chromogranin A release as a percentage of total cellular material. The same sample was also subjected to western blot analysis. After chemiluminescence development, slot blots were imaged with an iBRIGHT 1500 (Thermo Fisher Scientific) unit ordinarily in (4 × 4) bin mode. Images were saved as TIFF files and quantified in ImageJ. For presentation, images were adjusted for brightness and contrast with ImageJ “auto” function. A graph was plotted by using GraphPad Prism v10.1.2.

### Carbachol treatment

BON cells were seeded on poly-D-lysine-coated coverslips and co-transfected with mCherry-Munc13-4 or mCherry-Munc13-4 C2A*B* and CgA-EGFP constructs. Cells were incubated at 37°C for 15 minutes with LysoView^™^ 405 (1X; Biotium #70066) in culture medium to label lysosomes. Following incubation, cells were stimulated with carbachol (1 mM) and immediately imaged using Nikon A1R + confocal microscope.

### Ca^2+^ chelator treatment

BON cells were pre-treated for 30 minutes with either DMSO, BAPTA-AM (10 μM, Cayman #15551) or EGTA-AM (10 μM, Cayman #20401), followed by LI treatment for 3 hours. Cells were then harvested and analyzed for CgB and tubulin protein levels using Western blotting.

### Starvation

For macroautophagy studies, cells were briefly washed in serum-free DMEM and the media was aspirated. Cells were starved in a live cell imaging solution (140 mM NaCl, 20 mM HEPES, 2.5 mM KCl, 1.8 mM CaCl₂, and 1.0 mM MgCl₂, pH 7.4, with 1% FBS) with lysosomal inhibitors cocktails (Pepstatin A (Sigma #P5318) and E64D (Cayman # 13533) for 6 hours at 37°C.

### Structured illumination microscopy

Cells were seeded in 25 mM poly-D-lysine coated coverslips (Electron Microscopy Science #72290–12). Phogrin-EGFP and NPY-tdtomato constructs were transfected using Lipofectamine 3000. Cells were treated with lysosomal inhibitor (Pepstatin A (Sigma #P5318) and E64D (Cayman # 13533), and Bafilomycin (Sigma #196000) and fixed with 4% paraformaldehyde and LAMP1 antibody was used to stain lysosomes. Cells were mounted on coverslips in the presence of a slow fade mounting solution (Thermo Scientific #S36936). The SIM images were acquired with a Nikon N SIM microscope (SR-APO TIRF NA 1.49 100× lens, Andor iXon 3 EM-CCD camera, and 488-, 561-, and 640-nm laser lines) using three-dimensional (3D) SIM mode and reconstructed with NIS software. Images were then sharpened and contrast was adjusted using Image J.

### Endosomal SNAREs and Munc13-4 purification

pET28a plasmids that expressed VAMP-2, syntaxin-7, syntaxin-8, and Vti1b all tagged with a C-terminal His tag was the gift of J. Shen (University of Colorado, Boulder, CO). The proteins were purified using Ni-NTA (Qiagen) chromatography as described^46^. Additionally, the His6-tagged human Munc13-4 protein generated in Sf9 insect cells was purified using Ni-NTA agarose (Qiagen #30210) and further purified by Mono Q anion exchange chromatography (GE Healthcare #71-5017-90 AE), as previously described^45^.

### Proteoliposome preparation

1-Palmitoyl-2-oleoyl phosphatidylcholine (POPC), 1,2-dioleoyl phosphatidylserine (DOPS), 1,2-dioleoyl phosphatidylethanolamine (DOPE), and cholesterol were purchased from Avanti Polar Lipids. DiI and [^3^H]1,2-dipalmitoyl PC were obtained from Thermo Fisher Scientific and American Radiolabeled Chemicals, respectively, and used for lipid recovery and liposome-binding standardization. Lipid mixtures mimicked late endosomal composition as described^46^.

For Q-SNARE reconstitution, lipids (POPC, DOPE, DOPS, cholesterol, and DiI) were mixed at a 58:20:10:10:2 mol% ratio, dried under argon, and rehydrated with SNARE proteins in an elution buffer containing HEPES, KCl, imidazole, and β-octylglucoside. Proteoliposomes were generated by co-micellization at a protein:lipid ratio of 1:500, followed by overnight detergent removal through dialysis with SM-2 Biobeads. Proteoliposomes were purified via Accudenz gradient flotation and desalting using Zeba Spin columns.

### Single-step organelle enrichment

SGs were enriched from BON cells transfected with Phogrin-EGFP-twin-Strep, or lysosomes were isolated from stable BON cell lines expressing LAMP1-mScarlet-I-twin-Strep. All procedures were performed at 4°C using ice-cold reagents. Cells from 3–5 confluent 10 cm dishes were scraped into PBS, pelleted by centrifugation, and washed with homogenization buffer (0.26 M sucrose, 5 mM MOPS, 0.2 mM EDTA, 1 mM DTT). Following resuspension in homogenization buffer containing protease inhibitors, cells were lysed using a ball-bearing homogenizer. Post-nuclear supernatant (PNS) was obtained by centrifugation at 1,000 × g for 2 min. Mitochondria were removed by centrifugation at 8,000 × g for 15 min and incubated on ice. For affinity capture, 800 μl of PNS was incubated with 80 μl of pre-washed Strep-Tactin^®^ Sepharose^®^ resin (cat. 2-1201-002, IBA Lifesciences) in 1.5 ml microcentrifuge tubes. The mixture was gently rotated for 30 min at 4°C to allow binding of twin-Strep–tagged organelles. Beads were then washed three times with ice-cold homogenization buffer. SGs bound *via* Phogrin-EGFP-twin-Strep were eluted using 20 mM desthiobiotin in homogenization buffer. Lysosomes captured with StrepTactin beads were cleaved by overnight incubation with TEV Protease, biotin-tagged (# SAE0118, Sigma-Aldrich), at 4°C. The supernatant was then collected for downstream analysis.

### Mice

All mice used in this study and the surgical protocols were approved by the University of Wisconsin Institutional Animal Care and Use Committee (IACUC) and were carried out in accordance with IACUC guidelines and relevant regulations. The study is reported in accordance with the ARRIVE guidelines. Complete animal care facilities are provided by the UW Research Animal Resources Center (RARC). RARC personnel provide basic care, and veterinary care is available on site. The facility provides temperature- and light-controlled housing for animals, which is operated under the supervision of a full-time veterinarian. For the procedures described, mice weighed on average 22–28 g at the time of experimentation. Anesthesia was performed using isoflurane and euthanasia was carried out by CO_2_ asphyxiation and cervical dislocation in accordance with approved protocols.

Munc13-4 floxed mice were a gift from Roberto Adachi (Department of Pulmonary Medicine, The University of Texas MD Anderson Cancer Center). To generate global Munc13-4 knockout mice, the floxed line was crossed with B6.C-Tg(CMV-cre)1Cgn/J mice (The Jackson Laboratory; catalog no. 006054) that express Cre recombinase ubiquitously to delete the floxed allele in the germline and generate our global knockout line. Wild-type C57BL/6J mice served as controls^80^. Genomic DNA was extracted from mouse ear clips using the Qiagen DNA Extraction kit (#69504). Knockout was validated by PCR using the following primers: P1 5′-ATCCCAGATCAAAATGCTCCCAC-3′, P2 5′-GGAAAGGTGTGTCGCCATGGTG-3′, and P3 5′-CCAACATAAGGCTCTCTGAAGG-3′. P1 and P2 were used to differentiate between WT (+; 636 bp) and KO (no band) and P2 and P3 between the wildtype and hetero (− or +, respectively; 1.2 kb & 415 bp) and WT (+; 1.2 kb) alleles. PCR was set up using Terra^™^ PCR Direct Polymerase Mix (#639270). All mice were kept in a pathogen-free facility at the UW-Madison Animal Facility and handled by the Research Animal Resources and Compliance at the University of Wisconsin-Madison. Knockout was further confirmed by extracting tissue proteins and subjecting them to Western blotting.

### Isolation of mouse pancreatic islets.

Islets were extracted from mice through a collagenase digestion procedure, incorporating the following adjustments^79^. A solution of 0.6 mg/ml Type XI collagenase (Sigma-Aldrich #C7657) in HBSS (Thermo Fisher Scientific #14025092) supplemented with 0.35 g/l sodium bicarbonate and 0.02% RIA grade BSA (Sigma-Aldrich #126593) was injected into the common bile duct. The pancreas was excised and subjected to a 16-minute incubation at 37°C in a shaking water bath. Following two washes, the digested pancreas was filtered through a 1,000-mm mesh and underwent Ficoll (Sigma-Aldrich #F8016) gradient separation. For the ultimate purification step, islets were manually selected in HEPES Krebs buffer (20 mM HEPES, pH 7.4; 119 mM NaCl; 4.75 mM KCl; 2.54 mM CaCl_2_; 1.2 mM MgSO_4_; 1.18 mM KH_2_PO_4_; 5 mM NaHCO_3_) containing 5 mM glucose and 0.5% BSA. The brightfield illumination of the islets was assessed using a Leica MZ 95 dissection microscope at ×1, equipped with a cold light source (3,000 K). Cells were cultured in RPMI media with 5.5 mM glucose and fixed with paraformaldehyde for a week. Cells were immunostained for insulin, IA-2, and LAMP1 antibodies and imaged using Nikon A1R + confocal microscope. Images were deconvolved and brightness and contrast were adjusted using ImageJ. Co-localization was measured by Imaris Bitplane.

### Electron microscopy

BON cells and mouse β-cells were fixed with 2.5% glutaraldehyde and 2% paraformaldehyde. Post-fixation was performed using 1% osmium tetroxide and 1% potassium ferricyanide, followed by staining with saturated aqueous uranyl acetate. The cells were dehydrated with ethanol and propylene oxide, infiltrated, and embedded in Durcupan ACM resin. Thin sections were examined using a transmission electron microscope (CM120; Philips), and the number and size of SGs were analyzed using ImageJ. Mouse β-cells were cultured in RPMI medium containing 5.5 mM glucose for five days, washed with PBS. After staining with uranyl acetate and dehydration, they were embedded in Durcupan ACM resin. Sections were examined with TEM, and MATLAB’s Image Processing Toolbox, including the Image Segmenter tool, was used to quantify the electron micrographs.

### Lipid mixing fusion studies

To investigate docking and fusion between SGs and lysosomes, Strep-Tactin^®^ bead-immobilized SGs were incubated with lysosomes or SNARE-reconstituted liposomes for 15–30 min at 37°C. Fusion reactions were reconstituted using the presence and absence of 1 μM Munc13-4 in reconstitution buffer (25 mM HEPES, 140 mM potassium L-glutamate, 0.1 mM EGTA) with or without 24 μM [Ca^2+^]_f_. Following incubation, beads were washed three times with reconstitution buffer. Docked or fused organelles with the beads were then eluted using 20 mM desthiobiotin. 10 μL of the eluted sample was spread onto the glass cover slips and mounted on the glass slides for TIRF microscopy. Images were acquired using an iXon Ultra camera (Andor) and a 100×/1.49 NA ApoTIRF oil objective. Images were background-subtracted, and fluorescent objects were identified using the Spot module in Imaris (Bitplane). Colocalization was quantified by calculating the percentage of objects overlapping within a 250 nm distance. All image analysis was performed in a blinded manner, and thresholding parameters were applied uniformly across all experimental conditions to minimize bias.

### Co-immunoprecipitation

To investigate the interaction between various proteins either with 3xFlag or 3xHA Munc13-4, BON cells were co-transfected with GFP-tagged protein constructs. BON cells were lysed in ice-cold 1× lysis buffer (pH 7.5) containing 50 mM HEPES, 150 mM NaCl, 1% Triton X-100, cOmplete protease inhibitor cocktail (Roche Diagnostics #11873580001) on ice for 30 min, and further homogenized by passage through 10 μl tip fitted into 1ml tip ten times. The insoluble cell lysate fraction was removed by centrifugation at 10,000g and 4°C for 30 min to 1 hour. Cell lysates were further cleared by incubation with GFP selector (NanoTag Biotechnologies #N0310-L) for 30 min at 4°C followed by centrifugation at 1,000g and 4°C for 1 min. GFP agarose beads were pelleted by centrifugation at 1,000g and 4°C for 1 min and washed three times with an ice-cold 1× lysis buffer. Boiling agarose beads eluted pull-down proteins in 2× Laemmli sample buffer (Bio-Rad Laboratories #1610747) containing 5% β-mercaptoethanol (Sigma #M6250) for 5 min and then analyzed by western blot.

### In vitro protein binding

To investigate the interaction between Munc13-4 and PLEKHM1, HEK293FT cells (Invitrogen) were transfected with twin-Strep-tagged Munc13-4 and GFP-tagged PLEKHM1 constructs. After 48 hours, cells were lysed in Tris-NaCl buffer containing protease inhibitors. Lysates were cleared by centrifugation and processed using GFP selector beads for GFP-PLEKHM1 and Strep-Tactin^®^ Sepharose^®^ for Munc13-4 purification. Elution was performed with desthiobiotin, followed by desalting. Purified Munc13-4 was incubated with GFP nanobody-immobilized GFP or PLEKHM1 for interaction studies. After washing, proteins were eluted in Laemmli buffer, boiled, and analyzed by western blot.

### 3-color STORM imaging

Cells were seeded on poly-D-lysine-coated coverslips (Electron Microscopy Science #72290–12) and treated with lysosomal inhibitors (Pepstatin A and E64D) for 4 hours. After washing with PBS, fixation was performed using 3% PFA + 0.1% glutaraldehyde in 1 M PIPES (Sigma #P2949) for 15 minutes. Permeabilization was done with 0.2% Triton X-100 and 0.2% Saponin, followed by blocking with 3% BSA + 0.2% Triton X-100 in PBS. Primary and secondary antibodies (Alexa 488, Alexa 647, CF594^®^ST) were applied sequentially with washing steps. Post-fixation was done with 1.5% PFA. STORM imaging buffer contained glucose oxidase, catalase, MEA, and β-mercaptoethanol in a Tris-NaCl buffer. TetraSpeck^™^ Microspheres (Thermo Fisher #T7279) were used for calibration. Images were acquired (512×512 pixels, 19 ms/frame, 5,000 frames/channel) using an iXon Ultra camera (Andor) and a 100×/1.49 NA ApoTIRF oil objective. Molecule lists were generated with Nikon elements STORM software and analyzed with Huygens Localizer for cluster measurement. Data were graphed using GraphPad Prism.

### DQ Red-BSA content mixing assay.

Cells expressing CgA-EGFP were seeded onto 25 mm glass coverslips. Cells were loaded with DQ Red-BSA (Thermo Fisher Scientific # D12051) at a working concentration of 10 μg/ml in 1% FBS culture medium for 2 h −3 h at 37°C and 5% CO_2_. Cells were washed twice with PBS and imaged using live-cell imaging buffer (50 mM HEPES, 140 mM NaCl, 2.8 mM KCl, 1.8 mM CaCl_2_, 1mM MgCl_2_, and 5.5 mM glucose), and images were captured using Nikon A1R + confocal microscope. MatCol^81^ was used to quantify colocalization in images.

### TIRF microscopy and image analysis

BON-EGFP stable cells were seeded on poly-D-lysine coated coverslips (Electron Microscopy Sciences #72290–12). Cells were transfected with either mCherry-Munc13-4 WT or mCherry-Munc13-4 C2A*B* constructs 16 hours before the TIRF microscopy experiment. Immediately before imaging, cells were briefly washed with Live Cell Imaging Solution (Thermo Fisher Scientific #A59688DJ) supplemented with 10 mM glucose (Sigma #G8270) and 1% FBS (Gibco #16000044). Live cells were then imaged using total internal reflection fluorescence (TIRF) microscopy. Images were then sharpened and contrast was adjusted using Image J.

### Statistical analysis

We employed statistical methods to analyze the data. Two-sided Student’s unpaired t-tests were used to compare means between two independent groups assessing significant differences between their means. A one-way ANOVA was applied for comparing three or more groups to determine statistically significant differences. When the assumption of equal variances was unmet, Welch and Brown-Forsythe ANOVA tests were used to adjust for unequal variances. All data are presented as scatter dot plots with mean values and standard deviations (SD).

## Supplementary Material

Supplementary Files

This is a list of supplementary files associated with this preprint. Click to download.

- Supplementarytables.pdf
- Supplementaryinformation.pdf

## Figures and Tables

**Figure 8:**
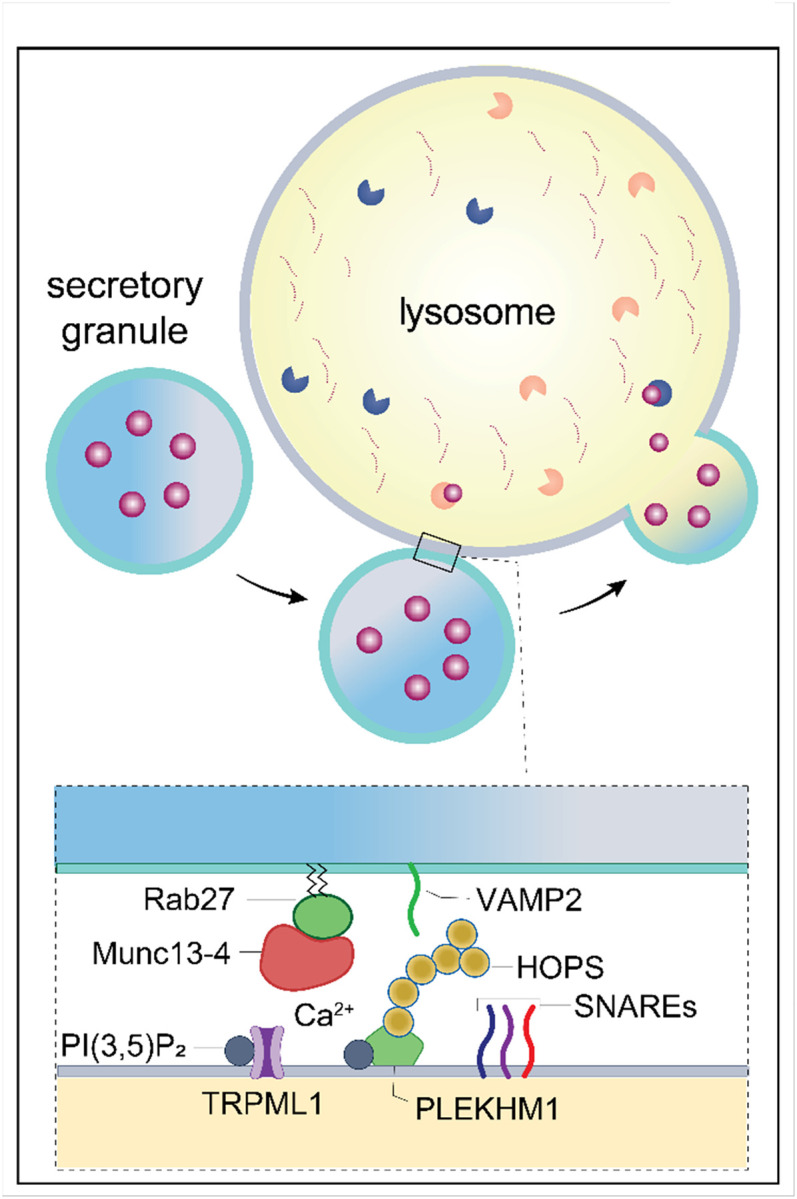
Proposed model for regulated crinophagy in mammalian endocrine cells. Crinophagy ensures the turnover of peptide hormone-containing SGs in resting endocrine cells by targeting older unused SGs for degradation *via* SG-lysosomal fusion, a process orchestrated by Munc13-4. siRNA and protein interaction screening showed that Munc13-4 on SGs bound to Rab27A plays a central role by coordinating interactions of the SG SNARE VAMP2 with lysosomal SNAREs STX8, STX7, VTI1B (shown schematically). Munc13-4 also interacts directly with the lysosomal tethering protein PLEKHM1 and possibly indirectly with the HOPS complex to enable docking and fusion. The fusion process is regulated by Ca^2+^ that binds to C2 domains of Munc13-4 to promote membrane fusion. Ca^2+^ could be global cytoplasmic increases or local lysosomal fluxes via PI(3,5)P_2_-dependent TRPML1 channels. In the absence of Munc13-4, SGs fail to fuse with lysosomes leading to impaired SG content.

## Data Availability

Primary data supporting this manuscript are available from the corresponding author upon request. **Antibodies**. All antibodies used in this study are listed in Supplementary Table 1. **Plasmids**. Plasmids used in this study are listed in Supplementary Table 2. **siRNA sequence information**. siRNA pools used in this study are listed in Supplementary Data S1.
